# Dysregulation of Caveolin-1 Phosphorylation and Nuclear Translocation Is Associated with Senescence Onset

**DOI:** 10.3390/cells10112939

**Published:** 2021-10-28

**Authors:** Andreas Goutas, Zozo Outskouni, Ioanna Papathanasiou, Maria Satra, George Koliakos, Varvara Trachana

**Affiliations:** 1 Department of Biology, Faculty of Medicine, School of Health Sciences, University of Thessaly, Biopolis, 41500 Larissa, Greece; agoutas@uth.gr (A.G.); zooutskouni@uth.gr (Z.O.); iopapat@med.uth.gr (I.P.); msatra@med.uth.gr (M.S.); 2 Biohellenika, Biotechnology Company, 57001 Thessaloniki, Greece; koliakos@med.auth.gr; 3 Department of Biological Chemistry, Medical School, Aristotle University of Thessaloniki, 54124 Thessaloniki, Greece

**Keywords:** caveolin-1, mesenchymal stem cells, oxidative stress, senescence, DNA damage

## Abstract

We recently reported that the inability of osteoarthritic (OA) chondrocytes to repair oxidative stress (OS) induced DNA damage is linked to Cav-1 overexpression/improper localization. We speculated that the senescent status of OA cells was responsible for this Cav-1 dysregulation. Here, to further investigate this hypothesis, we used Wharton Jelly derived mesenchymal stem cells (WJ-MSCs) and investigated Cav-1 function as cells reached replicative senescence or upon stress induced senescence (SIPS). We showed that Cav-1 is upregulated, phosphorylated and translocated to the nucleus in young WJ-MSCs upon acute exogenous OS, and that it returns back to basal/nonphosphorylated levels and exports the nucleus in the recovery phase. However, as cells reach senescence, this regulation is lost. OS did not induce any Cav-1-mediated response, which is concomitant with the inability of older cells to restore DNA damage. Furthermore, downregulation of Cav-1 resulted in persistent OS-induced DNA damage and subsequent onset of senescence. We also report that the establishment of senescence is mediated by autophagy stimulation, since downregulation of autophagy key molecule Atg5, simultaneously with Cav-1 downregulation, was found to inhibit SIPS. Basically, we propose that Cav-1 involvement in DNA damage response can lead to senescence, either because the damage is extensive or because Cav-1 is absent/unable to perform its homeostatic role.

## 1. Introduction

Mesenchymal stromal/stem cells (MSCs) have been defined as a heterogeneous population of cells which is characterized by its self-renewal capacity and ability to differentiate into a variety of mesodermal cell types (stemness) [1]. MSCs can be sourced from different tissues including bone marrow, adipose tissue as well as umbilical cord; it has been demonstrated that their functional characteristics are influenced by the source from which they are taken [2]. The umbilical cord, and especially its connective tissue, called Wharton’s jelly, is a rich source of MSCs (WJ-MSCs) that have a rapid growth rate, strong differentiation ability, and lower immunogenicity compared to MSCs isolated from other sources [3]. Moreover, they show fibroblastic morphology and have a three-lineage differentiation potential, i.e., adipogenic, osteogenic, and chondrogenic, indicating that WJ-MSCs are a potent candidate for regenerative medicine applications [4].

MSCs are considered to be a promising treatment for rare and intractable diseases, as these cells do not cause immune reactions when transplanted, and have demonstrated therapeutic efficacy in various diseases through their paracrine action [5,6]. Currently, the underlying mechanism(s) of the involvement of MSCs in tissue repair, such as the regenerative MSC secretome that includes the production of cytokine and paracrine factors, are an area of active discussion in the scientific literature [7]. The limited number of MSCs isolated from in vivo sources is a significant drawback of MSC-based therapy in the field of regeneration medicine and immunotherapy, giving rise to the need to obtain a large quantity of ex vivo culture-expanded MSCs [6]. However, MSCs start losing their proliferative potential as with increasing passage numbers, since they enter senescence after long term in vitro expansion [8].

Senescence, and especially stem cell senescence, plays a crucial role in tissue remodeling, wound healing as well as in tumorigenesis [9], and is also associated with aging-associated degenerative diseases [10]. Senescent cells exhibit distinctive morphological changes that include flattened appearance, higher granularity, enlargement in size and changes in the ratio of nucleus to cytoplasm [11]. They are basically nonreplicative, metabolically active cells that acquire a senescence-associated secretory phenotype (SASP) which is characterized by the secretion of specific cytokines, chemokines, and growth factors [9]. Different factors are known to induce cellular senescence, including telomere erosion, DNA damage accumulation, and mitochondrial dysfunction [12]. Senescence is classically divided into replicative senescence (RS) and stress-induced premature senescence (SIPS), but both present the morphological and phenotypic changes described above [13]. Oxidative stress (OS) is considered one of the main extrinsic stressors leading to MSC senescence [14]. Early passage (young) MSCs have low levels of reactive oxygen species (ROS) and high content of glutathione, a major cellular antioxidant. However, as they are expanded ex vivo, late-passage (old) MSCs have elevated ROS production which inhibits their proliferation [15]. Elevated OS may dysregulate DNA damage repair mechanisms, leading to DNA damage accumulation and subsequent loss of differentiation and self-renewal capability of MSCs [12]. In addition to dysregulated DNA damage repair mechanisms, OS and DNA damage seems to affect the autophagy function, which may also contribute to MSC senescence by inducing SASP [16].

In recent decades, growing evidence has supported the hypothesis that altered endocytic pathways promote cellular senescence. Caveolae-mediated endocytosis is considered one of the two major types of endocytosis involved in cellular senescence via multiple mechanisms [17]. Caveolae are 50–100-nm, flask-shaped invaginations of the plasma membrane enriched in cholesterol and glycosphingolipids [18,19]. Caveolin-1 (Cav-1) is a 21- to 24-kDa protein that is required for the formation of caveolae in most cell types [20]. Cav-1 has been extensively investigated in many biochemical studies involving cell signaling [21], oxidative stress response [22], and DNA damage repair [23,24]. Upregulation of Cav-1 is evident in senescent cells, and is linked to several age-related diseases [25,26]; therefore, it is characterized as a “gatekeeper” of cellular senescence as well as an ageing biomarker [27].

Additionally, we have previously demonstrated that exogenously OS-provoked DNA damage in chondrocytes was accompanied by upregulation of Cav-1 levels and its translocation to the nucleus. Importantly, we also showed that failure of spatiotemporal regulation of Cav-1 was associated with cell inability to restore OS-induced double strand breaks (DSB), and possibly with the onset of cellular senescence [28]. However, a decrease in the levels of Cav-1 has been demonstrated in cardiomyocytes [29] and myoblasts [30] after oxidative stimulation. Furthermore, several recent articles have contradicted the aforementioned reports regarding the connection between Cav-1 upregulation and senescence onset, demonstrating instead that the downregulation of Cav-1 induces senescence [31,32,33,34]. Therefore, in the present study, we explored the function of Cav-1 during replicative senescence (RS) and SIPS of MSCs, in an attempt to provide necessary information that could fill the gap in knowledge resulting from the publications of controversial data regarding the role of Cav-1 in senescence.

## 2. Materials and Methods

### 2.1. Primary Cultures of Mesenchymal Stem Cells

MSCs were obtained from the Wharton Jelly of umbilical cords (WJ-MSCs) from term-gestation newborns after birth, having obtained consent from the parents (three different individuals, *n* = 3), as previously described [35]. Isolated WJ-MSCs were cultured, as reported previously [36], in Dulbecco’s modified Eagle’s medium DMEM high glucose with stable glutamine and sodium pyruvate (BioWest, Miami, FL, USA) plus 10% fetal bovine serum (FBS; Thermo Fisher Scientific, Waltham, MA, USA) and 1% penicillin-streptomycin (Thermo Fisher Scientific, Waltham, MA, USA) at 37 °C in a humidified atmosphere of 5% CO_2_. Cells from all three donors were maintained in culture in order to produce early- (passage (*p*) < 14), middle- (15 < *p* <40), and late-passage cells (*p* > 40) that were used for the experiments. The medium was changed twice a week and cells were passed when confluency was reached.

### 2.2. OS Treatment

For acute exogenous oxidative insult, 200,000 cells were seeded per well on a six-well plate and, at 65–70% confluence, were exposed to 300 μΜ H_2_O_2_ for 30 min in a serum-free medium. The medium was then replaced with fresh complete medium and cells were left to recover for different lengths of time (1 h, 3 h and 24 h recovery time for western blot analyses and 1 h and 24 h for fluorescent microscopy analysis).

To achieve stress induced premature senescence (SIPS) by oxidative stress, we followed a previously reported method [37,38] with some modifications. In detail, cells at around 65–70% confluence were treated with 400 μM of H_2_O_2_ for 2 h, and subsequently washed with phosphate buffer saline (PBS) twice and normal medium was added. After 1–3 days, cells were split in 1:2 ratio (passage 1—after treatment—SIPS p1). After 3–7 days, at 80% confluency, cells were split again (passage 2—after treatment SIPS p2) and seeded in six-well plates with coverslips for immunofluorescence analysis, or without coverslips for either protein extraction and western blot or senescence associated (SA)-β-gal staining.

### 2.3. Doxorubicin Treatment

Doxorubicin was used to treat WJ-MSCs in the early and late passages. Cells were treated with 0.2 μΜ Doxorubicin in complete medium for 24 h at 37 °C in a humidified atmosphere of 5% CO_2_.

### 2.4. siRNA Transfection

One day before transfection, early passage cells were seeded onto a six-well plate and cultured in growth medium without adibiotics. At transfection time, cells at 60–80% confluence were transfected with 50pMol siRNA against Cav-1 (Qiagen, MD, USA) or combined with 25pMol siRNA against ATG5 (#SC-41445, Santa Cruz Biotechnology Inc, Dallas, TX, USA), as well as with negative control (#SC-37007, Santa Cruz Biotechnology Inc, Dallas, TX, USA) for 72 h. Transfected cells with siRNA against Cav-1 were also exposed to the aforementioned acute exogenous OS or SIPS. The transfection was performed using Opti-MEM Mediun (Invitrogen, Life Technologies, Paisley, UK) and Lipofectamine-RNAiMAX reagent (Invitrogen, Life Technologies, Paisley, UK) according to the manufacturer’s protocol.

### 2.5. Total ROS/Superoxide Detection

WJ-MSCs (200,000) were seeded in each well of a six-well plate for 24 h. The ROS Inducer Pyocyanin (PYO) [39] was added to a final concentration of 200 μM for 30 min to one of the samples in order to serve as positive control. Oxidative stress was detected by staining with two fluorescent dyes from the ROS-ID^®^ Total ROS/Superoxide detection kit (ENZ-51010, Enzo, Farmingdale, NY, USA). The intensity of the green dye (total ROS detection reagent) represents the level of oxidative stress, and the intensity of the orange dye (superoxide detection reagent) provides exclusive detection of superoxide.

### 2.6. Protein Extraction and Western Blot Analyses

Total proteins from cells were extracted by lysing on ice with RIPA lysis buffer [10 mM Tris (pH 7.5), 150 mM NaCl, 1 % Triton X-100, 1% sodium deoxycholated, 0.1% SDS, 1 mM EDTA] supplemented with protease and phosphatase inhibitors cocktail (Thermo Fisher Scientific, Waltham, MA, USA) for 30 min. Cell lysates were then centrifuged at 12,000 rpm for 15 min at 4 °C, and supernatants were collected. Protein concentrations were determined using a Pierce^TM^ BCA Protein Assay kit (Thermo Fisher Scientific, Waltham, MA, USA), and 20 μg of total protein were separated in 10% sodium dodecyl sulfate–polyacrylamide gel electrophoresis gels (SDS–PAGE) followed by transferring to polyvinylidene fluoride (PVDF) membranes (Thermo Fisher Scientific, Waltham, MA, USA). Τhe membranes were blocked with 5% *w/v* nonfat dry milk in TBS/0.1%Tween^20^ or 5% BSA in PBS/0.1%Tween^20^ for 1 h at 4 °C and then incubated overnight at 4 °C with specific primary antibodies against Cav-1 (1:1000 dilution, Cell Signaling Technology, Caveolin-1 Rabbit #3251), p-Cav-1 (1:1000 dilution, Cell Signaling Technology, Phospho-Caveolin-1 Tyr14 Rabbit #3267), LC3A/B (1:1000 dilution, Cell Signaling Technology, LC3A/B Rabbit #4108), p62 (1:1000 dilution, Cell Signaling Technology, SQSTM1/p62 Mouse #88588), Beclin-1 (1:1000 dilution, Cell Signaling Technology, Beclin-1 Rabbit #3495), PI3K-CIII (1:1000 dilution, Cell Signaling Technology, PI3 Kinase Class III Rabbit #4263), ATG5 (1:1000 dilution, Cell Signaling Technology, Atg5 Rabbit #12994) ATG13 (1:1000 dilution, Cell Signaling Technology, Atg13 Rabbit #13468) ULK1 (1:1000 dilution, Cell Signaling Technology, ULK1 Rabbit #8054). Membranes were further probed with an antibody against β-actin (1:3000 dilution, Mouse, Santa Cruz Biotechnology Inc.), which served as a loading control. In subcellular fractionation analyses, antibody against alpha-Tubulin (1:1000 dilution, Cell Signaling Technology, alpha-Tubulin Rabbit #2125) was used as loading control for cytoplasmic fractions and antibody against Histone H3 (1:1000 dilution, Abcam ab1791) as loading control for nuclear fractions. Subsequently, membranes were washed three times with TBS/0.1%Tween^20^ for 10 min and then incubated with the appropriate horseradish peroxidase (HRP)-conjugated secondary antibodies for 1 h at room temperature (RT) [Antirabbit; 1:10,000, Invitrogen, Life Technologies, Paisley, UK, Antirabbit; 1:10,000, #BA1054-1, Boster, CA, USA, and antimouse; 1:10,000, #BA1050-1, Boster, CA, USA]. All protein bands were visualized using ECL substrates (Thermo Fisher Scientific, Waltham, MA, USA) and detected by Uvitec Cambridge Chemiluminescence Imaging System. The protein expression was quantified using Image J software (1.47r, Wayne Rasband National Institutes of Health, USA, https://imagej.nih.gov/ij/, accessed on 25 October 2021). Each western blot analysis was performed at least three times.

### 2.7. Immunofluorescence

Immunofluorescence experiments were performed as previously described [40] with slight modifications. In particular, 100,000 cells grown on coverslips in six-well plates were fixed in ice cold absolute methanol for 10 min at −20 °C. After blocking in PBS containing 0.2% Tween^20^ and 1% BSA for 10 min, coverslips were incubated with primary antibody against Cav-1 (1:500, #A1915, rabbit polyclonal, Santa Cruz Biotechnology Inc.) for 1 h at RT, followed by incubation with appropriate fluorescent dye-conjugated secondary antibody for 1 h at RT (1:500 dilution, Alexa Fluor 594, Molecular Probes). For DSB detection, fixed slides were incubated with specific primary antibody against 53BP1 (1:500, clone BP13, mouse monoclonal, Millipore, MA, USA) and the corresponding secondary antibody (1:500 dilution, Alexa Fluor 488, Molecular Probes). Vectashield mounting medium (Vector Laboratories, Burlingame, CA, USA) containing 4,6-diamidino-2-phenylindole (DAPI) was used to visualize nuclei. Images were taken using ZEISS Axio Imager.Z2 fluorescent microscope and were analyzed with ImageJ software. At least five randomly selected fields were analyzed by two independent observers blinded to the origin of the sample (early- or late-passage cells) for each time point. The means of their counts were used for the statistical analysis.

### 2.8. Senescence-Associated β-Galactosidase (SA-β-gal) Staining

Cells were washed three times with cold PBS and then fixed with 2% formaldehyde/0.2% glutaraldehide for 5 min at RT. After fixation, the cells were washed three times with cold PBS and then stained with freshly prepared SA-β-gal staining solution (40 mM citric acid/sodium phosphate buffer pH 6.0, 5 mM potassium ferrocyanide, 5 mM potassium ferricyanide, 150 mM NaCl, 2 mM MgCl_2_, 1 mg/1 mL 5-bromo-4-chloro-3-indolyl-β-D-galactoside) overnight at 37 °C. At least 300 cells were counted by 2 independent researchers. The number of SA-β-gal positive cells was expressed as a percentage of all cells counted.

### 2.9. Subcellular Fractionation

WJ-MSCs at 65–70%% confluence were treated with 300 μM for 30 min in serum-free medium and left to recover for different time points as described above. Cells were then collected in 1.5 mL tube. Pelleted cells were resuspended in 150 μL ice-cold buffer A (10 mM HEPES-KOH pH 7.6 at 4 °C, 1.5 mM MgCl_2_, 10 mM KCl, 1 mM PMSF, 0.5 mM DTT supplemented with protease and phosphatase inhibitors cocktail (Thermo Fisher Scientific, Waltham, MA, USA) for 30 min on ice and vortexed for 10 sec every 10 min. After centrifugation (7000 rpm 3 min 4 °C) the supernatant was collected and stored at −20 °C (cytoplasmic protein fraction). Then 150 μL ice-cold buffer B was added to the pellet (20 mM HEPES-KOH pH 7.6 at 4 °C, 1.5 mM MgCl_2_, 420 mM NaCl, 0.2 Mm EDTA, 1 mM PMSF, 0.5 mM DTT 25% Glycerol) supplemented with protease and phosphatase inhibitors cocktail (Thermo Fisher Scientific, Waltham, MA, USA) for 30 min on ice and vortexed for 10 sec every 10 min. After centrifugation (7000 rpm, 3 min, 4 °C) the supernatant was collected and stored at −20 °C (nuclear protein fraction).

### 2.10. Statistical Analysis

The SPPS 24 software was used for data analyses. Statistical significance was determined using the Student *t*-test. For all comparisons, *p* values less than 0.05 were considered statistically significant, and the significance on the graphs is marked with hashes (#=*p* < 0.05) when comparisons were made between early and late cells, or with asterisks (*=*p* < 0.05) when comparisons were made between each time point versus the No treatment (NT) condition. Results are reported as mean ± standard error (means ± S.Ε).

## 3. Results

### 3.1. Cav-1 Levels Assessment as Cells Reach Senescence

**Figure 1:**
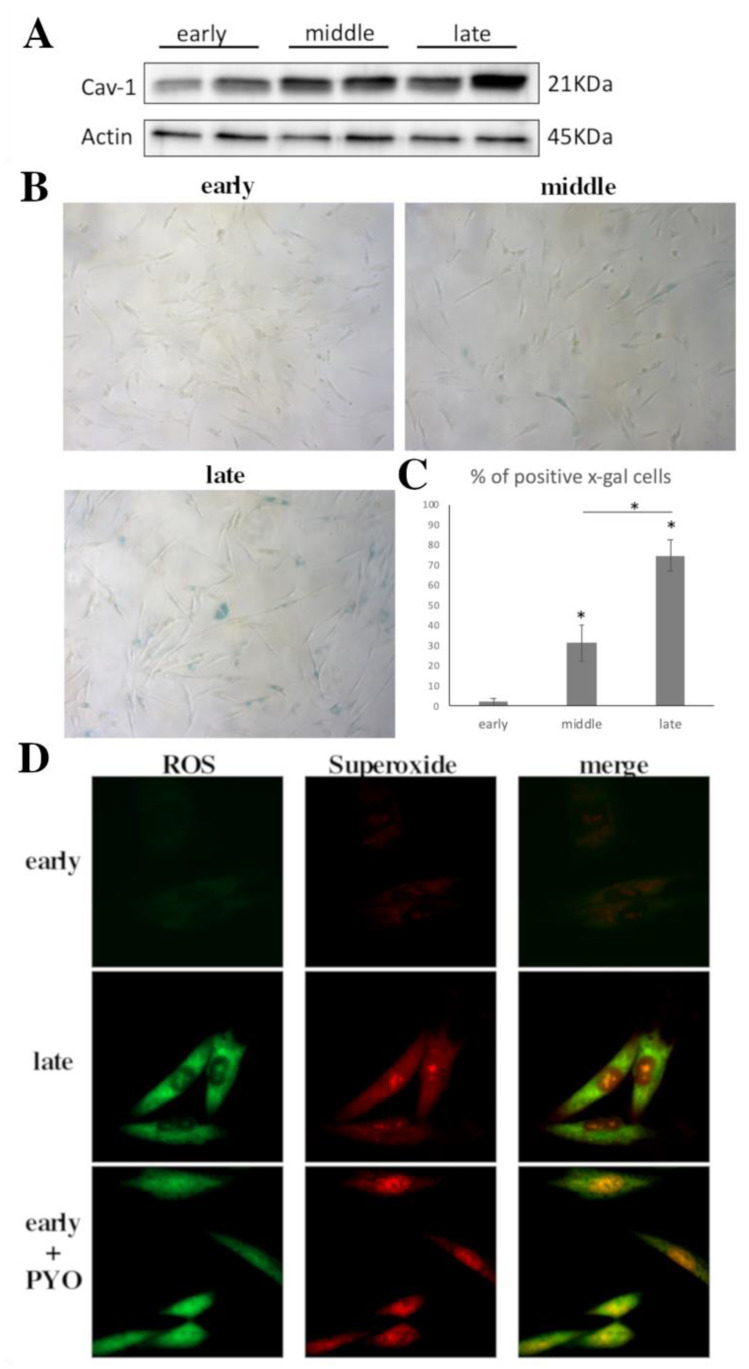
Cav-1 levels increase as cells reach senescence. (**A**) Characteristic Immunoblot showing caveolin-1 (Cav-1) levels increasing significantly as WJ-MSCs reach senescence. β-Actin was used as control for equal protein loading. (**B**) Representative photos of early-, middle- and late-passage cells stained with SA-β-gal staining solution. (**C**) Graph showing the percentage of SA-β-gal positive cells in early-, middle- and late-passage cells as mean from three donors. * *p* < 0.05 vs. NT or otherwise indicated. *p* values calculated using the Student *t*-test. (**D**) Representative images of early- and late-passage WJ-MSCs where total ROS and superoxide levels were analyzed under normal conditions (early–late). For a positive control pyocyanine was added to the early passage cells (early + PYO). Images were captured with the 40X objective lens of the fluorescent microscope used.

Cav-1 protein expression was analyzed by western blot in early- (passage (*p*) < 14), middle- (15 < *p* < 40) and late-passage cells *p* > 40). Figure 1A shows the differences in Cav-1 levels between the different passages of cultured WJ-MSCs. The band density revealed that Cav-1 protein levels were increased as cells aged (Figure 1A). Furthermore, this increase in Cav-1 levels was accompanied by an increased percentage of WJ-MSCs positive for senescence associated beta-galactosidase activity (SA-β-gal), which was indicative of cells establishing a senescent phenotype (Figure 1B) As shown in Figure 1C, the percentage of SA-β-gal positive cells increased from 2.2 ± 1.7 in early-passage to 31.3 ± 9. 9 in middle-passage and 74.6 ± 7.71 in late-passage cells. Moreover, as expected, the amount of oxidative stress increased as cells approach the senescent state, as indicated by the assessment of the amount of ROS and superoxide present in late-passage cells as compared to early passage cells (Figure 1D).

### 3.2. Cav-1 Regulation (Expression, Localization, Phosphorylation) and DNA Damage Assessment in Early- and Late-Passage WJ-MSCs after Exogenous Oxidative Insult

**Figure 2:**
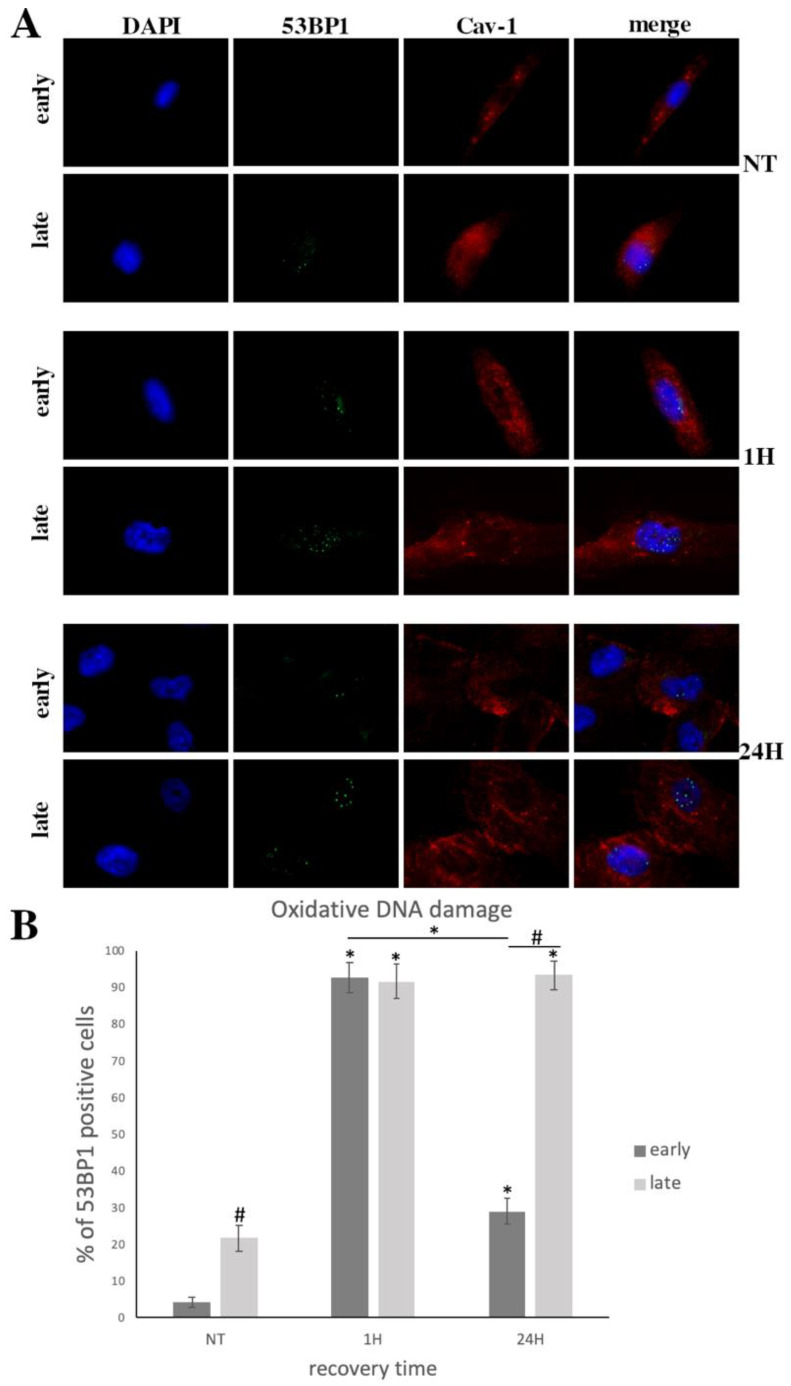
Late-passage cells demonstrated an inability to repair DNA damage. (**A**) Characteristic images of early- and late-passage cells where DNA damage was assessed by staining with the 53BP1 antibody and the appropriate secondary antibody (green). (blue). Cav-1 localization was also evaluated by staining with anti-cav-1 antibody and the appropriate secondary antibody (red) under normal conditions (No Treatment—NT) and at 1 h and 24 h after being treated with H_2_O_2_. (300 μΜ, 30 min). Nuclei were stained with DAPI. Images were captured with the 40× objective lens of the fluorescent microscope used. (**B**) Percentages of WJ-MSCs with 53BP1 foci from early- and late-passage cells from three donors evaluated by staining with the 53BP1 antibody under normal conditions (No Treatment—NT) and at different time points of the recovery period from the oxidative treatment (1 h and 24 h). Values shown are the means ± S.E. # *p* < 0.05 vs. early passage cells at the same time point and * *p* < 0.05 vs. NT or otherwise indicated. *p* values calculated using the Student *t*-test.

It has been previously reported that exposure to exogenous oxidative stress (OS) results in the formation of single strand breaks (SSB) which could turn into double strand breaks (DSB) in subsequent rounds of DNA replication [41]. In order to evaluate DSB formation in cultured early- and late-passage cells after exposure to exogenous oxidative insult (H_2_O_2_ 300 μΜ, 30 min), 53BP1 foci, that mark the presence of DSB [42], were microscopically assessed. As shown in Figure 2A,B a significantly larger number of cells with 53BP1-labelled lesions was observed in late-passage cells as compared to early passage cells, even before treatment (NT). The elevated oxidative status that we showed previously (Figure 1D) may have accounted for the OS-induced DNA lesions that were observed in late-passage cells as compared with younger cells under normal conditions.

Furthermore, at 1 h post-treatment with H_2_O_2_, both early- and late-passage cells showed a remarkable increase to similar levels of DSB. Importantly, 24 h post-treatment, a significant decrease in the levels of DSB was observed in young cells, while in late-passage cells, the amount of DSB remained unchanged. This result reaffirmed our previously reported data on DNA damage repair failure in older cells [36].

**Figure 3:**
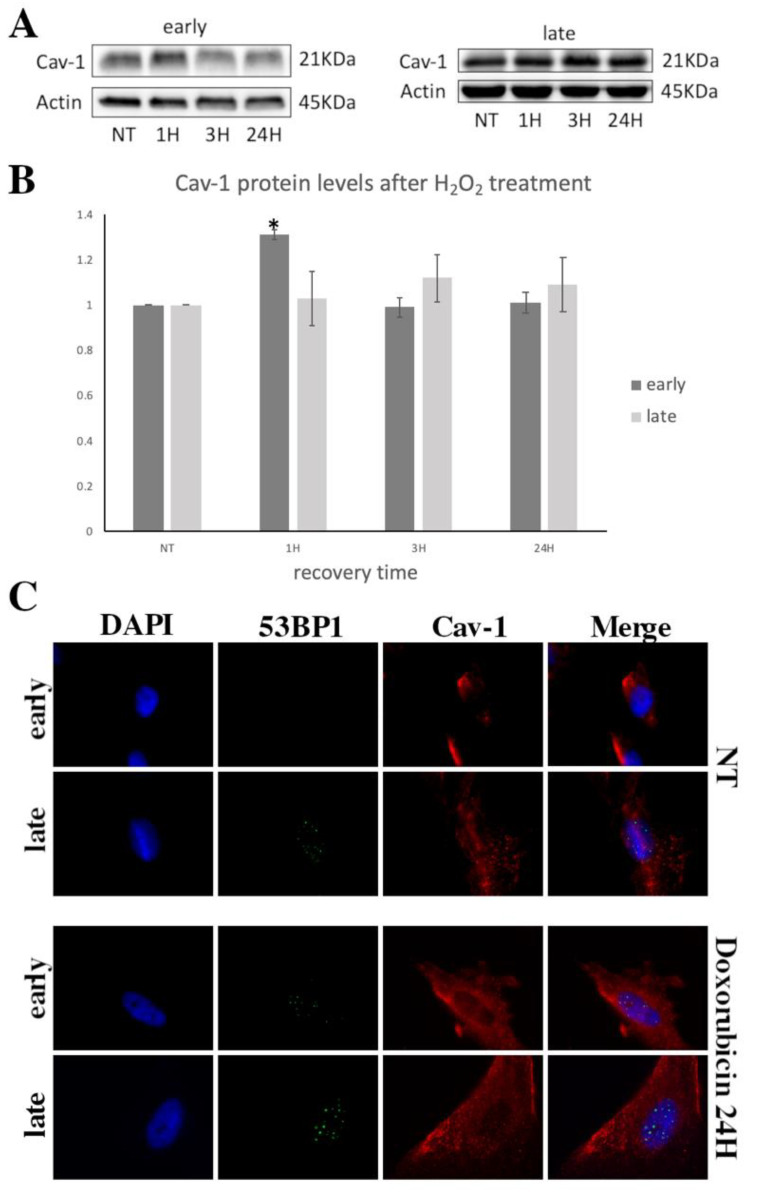

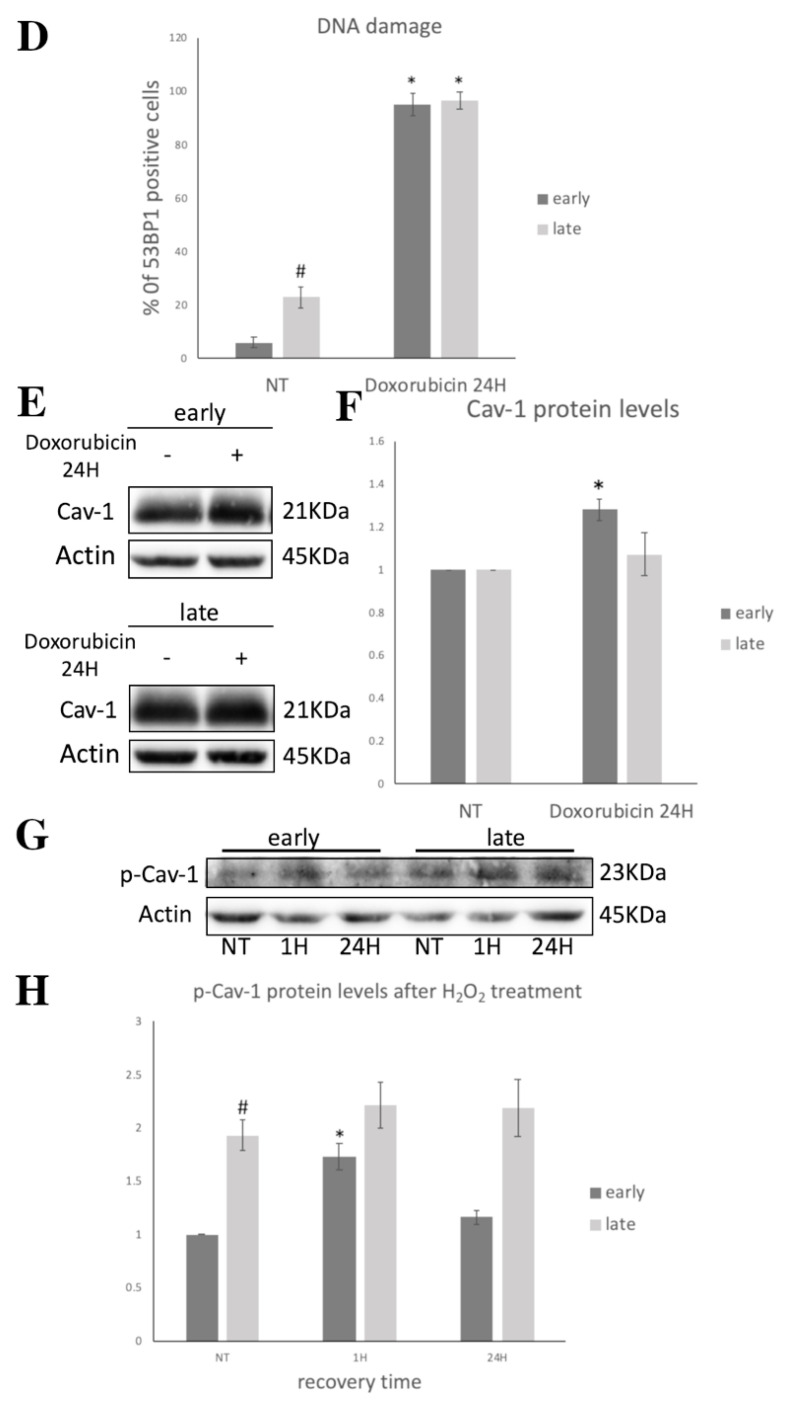
No alterations in Cav-1/pCav-1 levels in late-passage cells after exogenous oxidative insult. (**A**) Representative immunoblot of cell lysates of early- and late-passage WJ-MSCS (from three donors) that have been exposed to H_2_O_2_ (300 μΜ, 30 min) and left to recover for 24 h, analyzed at four different time points (NT(=No Treatment), 1 h, 3 h, and 24 h of recovery time post-treatment) by western blotting using the anti-caveolin-1 (Cav-1) antibody. (**B**) Quantification of relative protein expression levels of Cav-1 in cell lysates from early and late WJ-MSCs (from 3 donors) after being exposed to H_2_O_2_ was performed based on band density using ImageJ. NT values from both early- and late-passage cells were arbitrarily set to 1. Values shown are the means ± S.E. * *p* < 0.05 vs. the NT condition. (**C**) Characteristic images of early- and late-passage cells where Cav-1 localization was evaluated by staining with anti-cav-1 antibody and the appropriate secondary antibody (red) under normal conditions (No Treatment—NT) and 24 h after being treated with Doxorubicin. DNA damage was also assessed by staining with the 53BP1 antibody and the appropriate secondary antibody (green). Nuclei were stained with DAPI (blue). Images were captured with the 40× objective lens of the fluorescent microscope used. (**D**) Percentages of WJ-MSCs with 53BP1 foci from early- and late-passage cells from three donors evaluated by staining with the 53BP1 antibody under normal conditions (No Treatment—NT) and 24 h after Doxorubicin treatment. Values shown are the means ± S.E. # *p* < 0.05 vs. early passage cells at NT and * *p* < 0.05 vs. NT or otherwise indicated. *p* values calculated using the Student *t*-test. (**E**) Representative immunoblot of cell lysates of early- and late-passage WJ-MSCS (from 3 donors) that have been exposed for 24 h to Doxorubicin using anti-caveolin-1 (Cav-1) antibody. (**F**) Quantification of relative protein expression levels of Cav-1 in cell lysates from early and late WJ-MSCs (from 3 donors) after being exposed to 24 h Doxorubicin was performed based on band density using ImageJ. NT values from both early- and late-passage cells were arbitrarily set to 1. Values shown are the means ± S.E. * *p* < 0.05 vs. the NT condition. (**G**) Representative immunoblot showing p-Cav-1 levels from cell lysates of early- and late-passage WJ-MSCS (from three donors) that have been exposed to H_2_O_2_ (300 μΜ, 30 min) and left to recover for 24 h, analyzed at three different time points ((NT(=No Treatment), 1 h and 24 h of recovery time post-treatment). Antibody against β-Actin was used as loading control for all western blots (A, B, D). (**H**) Quantification of relative protein expression levels of p-Cav-1 in cell lysates from early and late WJ-MSCs (from three donors) after being exposed to H_2_O_2_ was performed based on band density using ImageJ. Values shown are the means ± S.E. * *p* < 0.05 vs. the NT condition and # *p* < 0.05 vs. early passage cells. *p* values were calculated using Student *t*-test.

The proteins levels of Cav-1 were also analyzed in response to exposure to exogenous oxidative stress. As shown in Figure 3A, in young cells, Cav-1 levels increased significantly 1 h post-treatment before returning to basal levels at 3 and 24 h after exposure to OS. In contrast, in older cells, there was no significant change in Cav-1 levels at any time point assessed (Figure 3A,B). Moreover, in order to examine if Cav-1 upregulation is due to oxidative stress or OS-induced DSB, we used Doxorubicin, a drug known to also induce DSB [43]. As shown in Figure 3C,D both early- and late-passage cells showed a remarkable increase to similar levels in 53BP1 staining after 24 h treatment. However, Cav-1 protein levels were found to be increased only in early passage cells after the treatment (Figure 3E,F), further implying the inability of late-passage cells to upregulate Cav-1 upon DSB formation.

Importantly, these differences between young and older cells were also evident when the levels of the phosphorylated form of Cav-1 were analyzed. As shown in Figure 3G,H, phosphorylated Cav-1 levels increased in young cells 1 h after the exposure to OS but returned to basal levels at 24 h, while in older cells, no differences were observed. The above implies an association between Cav-1 upregulation and phosphorylation in response to DNA damage in young cells which seems to be missing in older cells.

**Figure 4:**
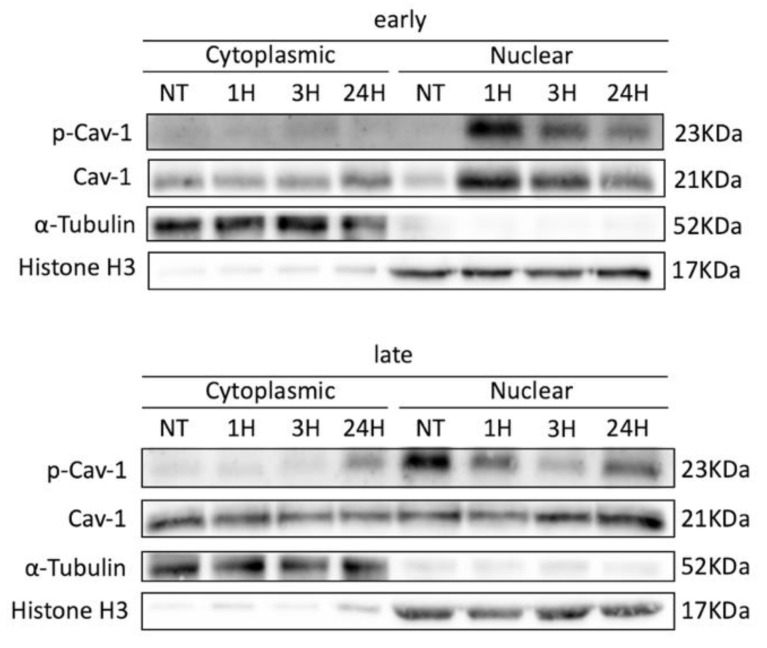
Cav-1 translocation to the nucleus depends upon its phosphorylation. Representative immunoblot of cell lysates of early- and late-passage WJ-MSCS that have been exposed to H_2_O_2_ (300 μΜ, 30 min) and left to recover for 24 h, analyzed at four different time points (NT(=No Treatment), 1 h, 3 h, and 24 h of recovery time post-treatment) after subcellular fractionation to cytoplasmic and nuclear fractions using the anti-caveolin-1 (Cav-1) and anti-Phospho-Caveolin-1 antibodies. Antibody against alpha-Tubulin was used as loading control for cytoplasmic fractions and antibody against Histone H3 as loading control for nuclear fractions.

Besides the differences observed between early- and late-passage cells regarding their ability to repair DNA damage (Figure 2A,B), microscopy analysis also demonstrated differences in the localization of Cav-1 between young and older cells (Figure 2A and Figure 3C). We previously analyzed the localization pattern of Cav-1 in normal and osteoarthritic (OA) chondrocytes and found that Cav-1, in cells derived from healthy individuals, was mainly found at the plasma membrane and cytoplasm, whereas in OA chondrocytes were found in the nucleus as well [28]. Given that OA chondrocytes are considered to be senescent cells [44], the current result did not come as a surprise: in late-passage WJ-MSCs, even before exposure to OS, Cav-1 was found to localize not only in the cytoplasm and plasma membrane, as in young cells, but also in the nucleus. In addition, in both early- and late-passage cells at 1 h post-treatment, Cav-1 was found to be localized in the nucleus, but after 24 h of recovery, in young cells only did it return to its original localization sites (Figure 2A). This result was further confirmed with western blot analysis after nuclear/cytoplasmic fractionation (Figure 4). Furthermore, the result regarding the phosphorylation status of the different fractions, combined with the result demonstrated in Figure 3 G,H, implies that Cav-1 is phosphorylated in response to OS, and that this could result in its translocation to the nucleus in early passage cells. In contrast, in late-passage cells, both Cav-1 total levels as well as its phosphorylated levels were elevated even before exposure to H_2_O_2_ (NT), implying a dysregulation in Cav-1 mediated OS response.

### 3.3. Cav-1 Levels Assesssment in SIPS

**Figure 5:**
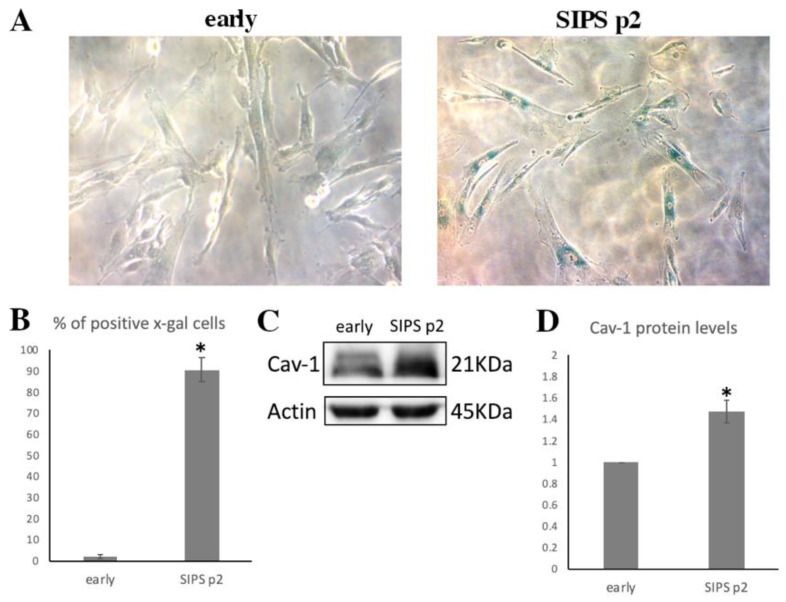
Cav-1 levels increase in SIPS. (**A**) Representative photos of early passage (early) cells untreated (early) and after being subjected to stress induced premature senescence (SIPS) by treatment with 400 μΜ of H_2_O_2_, 2 h at passage 2 (SIPS p2) stained with SA-β-gal staining solution. (**B**) Graph showing the percentage of SA-β-gal positive cells in early and SIPS p2 cells. Values shown are the means ± S.E. * *p* < 0.05 vs. early passage WJ-MSCs. (**C**) Characteristic immunoblot showing caveolin-1 (Cav-1) levels increasing significantly in SIPS p2 cells. Antibody against β-Actin was used as loading control. (**D**) Graph demonstrating Cav-1 levels in early and SIPS p2 cells, based on band density calculated using Image J. Values shown are the means ± S.E. * *p* < 0.05 vs. the early passage WJ-MSCs.

We opted for stress induced senescence (SIPS) in young WJ-MSCs by exposing them to oxidative stress in order to assess the Cav-1 function in this process. As described under “Materials and Methods”, early passage (*p* = 6) cells were exposed to 400 μΜ of H_2_O_2_ for 2 h and then normally cultivated for two additional passages. As shown in Figure 5A,B, cells at passage 2 (p2) after the above mentioned treatment showed the characteristic morphology of senescent cells, and 90.66 ± 5.5% of them were SA-β-gal positive. Moreover, when the levels of Cav-1 were analyzed in these cells, they were found to be significantly upregulated compared to early passage, untreated cells (Figure 5C,D).

### 3.4. Cav-1 Downregulation, DNA Damage Assessment and SIPS

**Figure 6:**
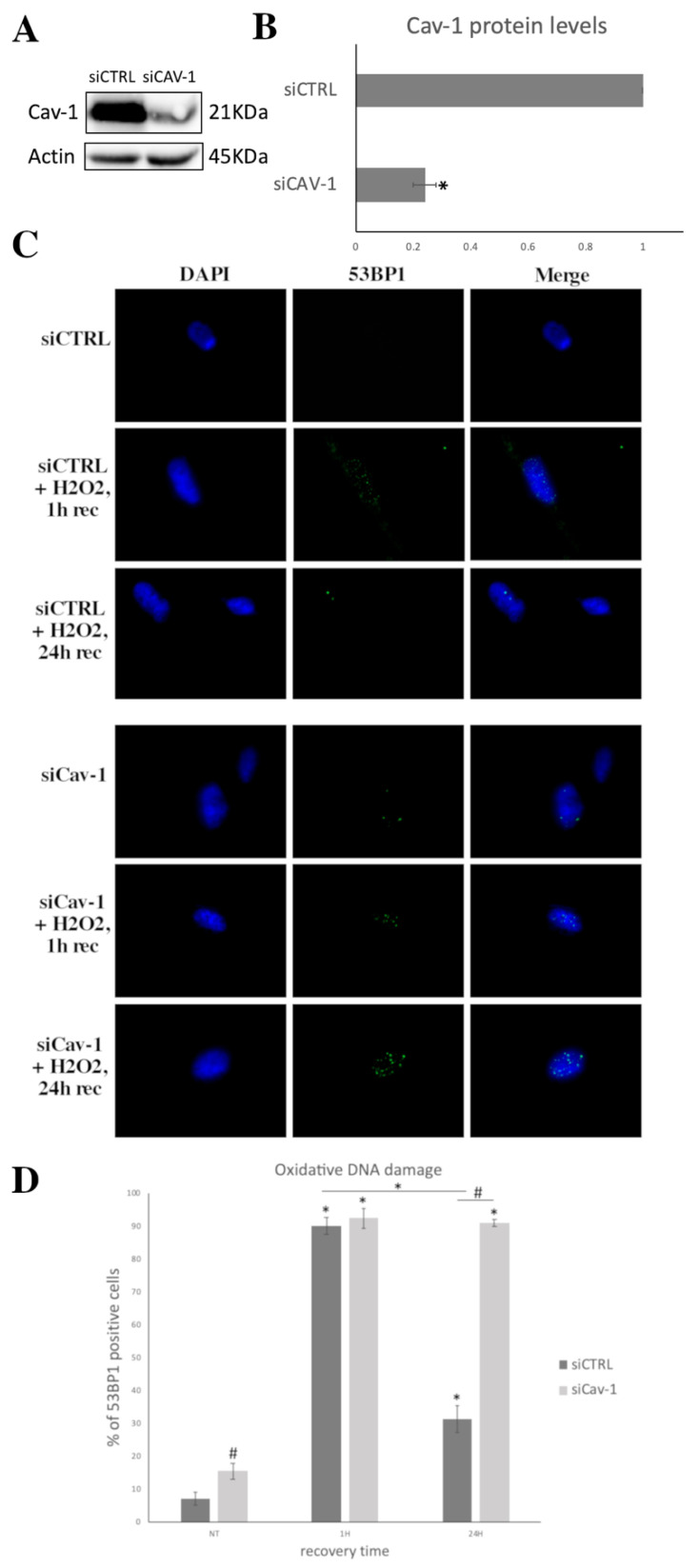
Cav-1 downregulation results in failure of oxidative DNA damage repair. (**A**) Characteristic immunoblot showing caveolin-1 (Cav-1) levels decreasing significantly after siRNA against Cav-1 treatment in early passage cells. Antibody against β-Actin was used as loading control. (**B**) Graph demonstrating Cav-1 levels based on band density calculated using Image J. Values shown are the means ± S.E. * *p* < 0.05 vs. the early passage WJ-MSCs treated with control siRNA (siCTRL). (**C**) Characteristic images of early passage cells treated with control siRNA and siRNA against Cav-1, under normal conditions (No Treatment—NT) and at 1 and 24 h after being treated with H_2_O_2_ (300 μΜ, 30 min) stained with anti-cav-1 antibody and the appropriate secondary antibody (red). DNA damage was also assessed by staining with the 53BP1 antibody and the appropriate secondary antibody (green). Nuclei were stained with DAPI (blue). Images were captured with the 40× objective lens of the fluorescent microscope used. (**D**) Percentages of cells with 53BP1 foci from early passage WJ-MSCs treated with siCTRL and siCav-1 under normal conditions (No Treatment—NT) and at 1 h and 24 h after being treated with H_2_O_2_ (300 μΜ, 30 min). Values shown are the means ± S.E. # *p* < 0.05 vs. siCTRL treated early passage cells and * *p* < 0.05 vs. NT or otherwise indicated. *p* values calculated using the Student *t*-test.

Given all of the above observations, we opted to downregulate Cav-1 levels by treating with a specific siRNA against Cav-1 early passage cells and subsequently analyzing the levels of DSB after exposure to OS. As shown in Figure 6A,B, when cells were treated with siRNA against Cav-1, its levels were dramatically decreased. Regarding DSB assessment, as expected, in control siRNA treated WJ-MSCs, the levels of 53BP1 dramatically increased 1 h after treatment with H_2_O_2_ (300 μΜ, 30 min) and decreased to significantly lower levels after 24 h of recovery. In contrast, young cells treated with siRNA against Cav-1 seemed unable to recover from the OS, as the levels of DSB reached 92.33 ± 3.05% 1 h post-treatment and remained almost unchanged (91 ± 1%) even 24 h after OS insult (Figure 6C,D).

**Figure 7:**
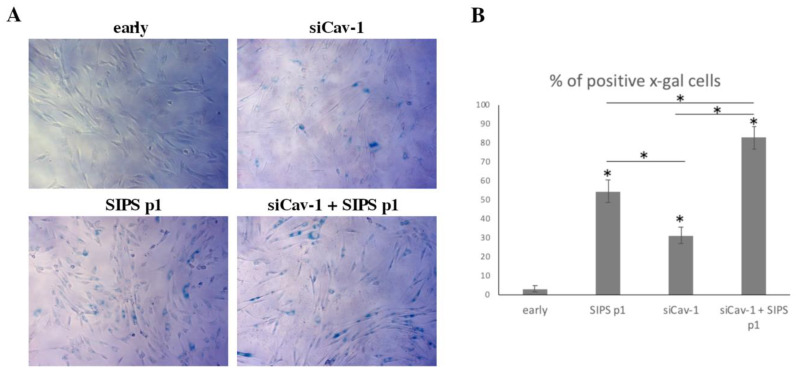
Cav-1 downregulation results in accelerated SIPS. (**A**) Representative photos of early passage WJ-MSCs (early), early passage WJ-MSCs treated with siRNA against caveolin-1 (siCav-1), early passage cells after being subjected to stress induced premature senescence (SIPS) at passage 1 (SIPS p1) and early passage WJ-MSCs treated with siRNA against caveolin-1 after being subjected to stress induced premature senescence (SIPS) at passage 1 (siCav-1+SIPS p1), stained with SA-β-gal staining solution. (**B**) Graph showing the percentage of SA-β-gal positive cells in early, SIPS p1, siCav-1 and siCav-1+SIPS p1 WJ-MSCs. *p* values shown are the means ± S.E. * *p* < 0.05 vs. early passage or otherwise indicated. *p* values calculated using the Student *t*-test.

In order to explore the role that Cav-1 plays in inducing senescence in response to DNA damage, early passage cells in which Cav-1 levels were downregulated using siRNA were exposed to H_2_O_2_ in order to induce SIPS, as described previously (see Figure 5). As shown in Figure 7, at passage 1, after the induction of SIPS, 54.3 ± 5.95% of control cells were SA-β-gal positive. Interestingly, the siRNA against Cav-1 without any other treatment also induced senescence at early passage cells (31 ± 4.39% SA-β-gal positive). Importantly, when siRNA against Cav-1 was combined with SIPS treatment, a significantly higher percentage (82.5 ± 6.08%) of the cells seemed to have reached senescence, as compared to individual treatments. The latter strengthens the assumption of the involvement of Cav-1 in inducing SIPS, possibly via its involvement in DNA damage repair.

### 3.5. Cav-1 Downregulation and Autophagy Assessment

**Figure 8:**
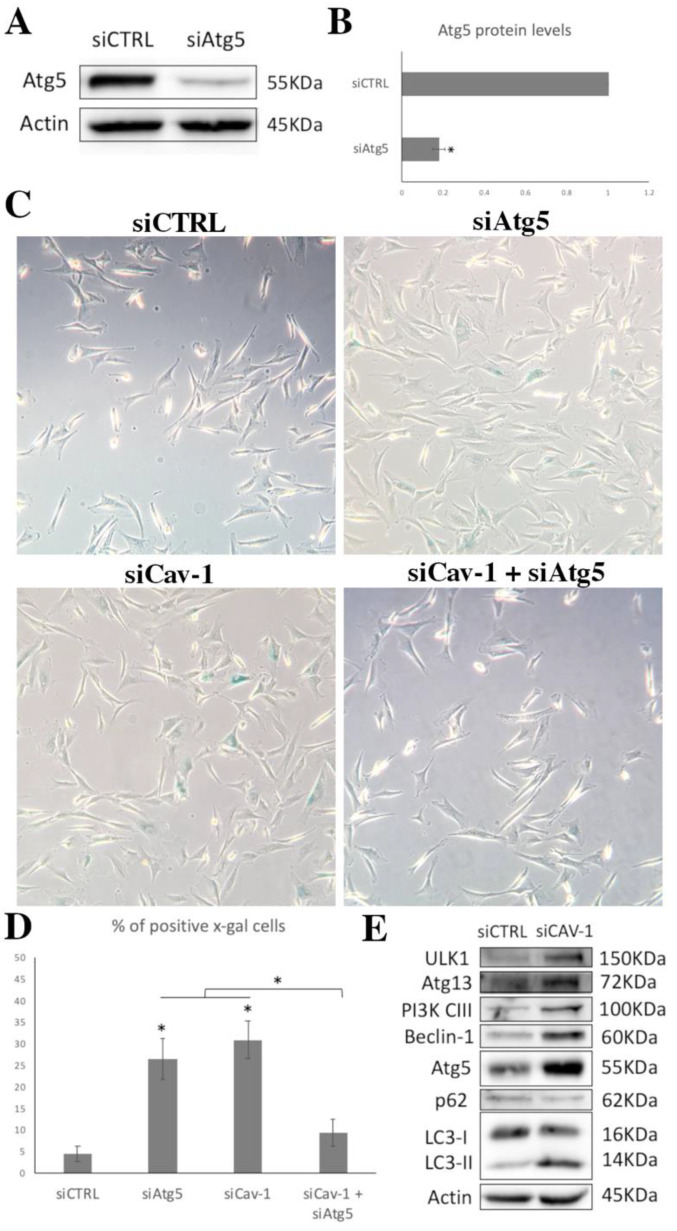
Cav-1 function in senescence is mediated by autophagy. (**A**) Characteristic immunoblot showing Atg5 levels decreasing significantly after siRNA against Atg5 treatment in early passage cells. Antibody against β-Actin was used as loading control. (**B**) Graph demonstrating Atg5 levels based on band density calculated using Image J. Values shown are the means ± S.E. * *p* < 0.05 vs. the early passage WJ-MSCs treated with control siRNA (siCTRL). (**C**) Representative photos of early passage WJ-MSCs treated with siRNA control (siCTRL), siRNA against Atg5 (siAtg5), siRNA against caveolin-1 (siCav-1), and early passage WJ-MSCs treated with double siRNA against caveolin-1 (siCav-1) and against siAtg5 (siCav-1+siAtg5) stained with SA-β-gal staining solution. (**D**) Graph showing the percentage of SA-β-gal positive cells in early passage WJ-MSCs treated with siRNA control (siCTRL), siRNA against Atg5 (siAtg5), siRNA against caveolin-1 (siCav-1), and early passage WJ-MSCs treated with double siRNA against caveolin-1 (siCav-1) and against siAtg5 (siCav-1+siAtg5). *p* values shown are the means ± S.E. * *p* < 0.05 vs. siCav-1. (**E**) Characteristic immunoblot showing protein levels of key molecules of autophagy (ULK1, Atg13, PI3K-III, Beclin-1, Atg5, p62, LC3-I/II) in early passage cells treated with control siRNA (siCTRL) or siRNA against Cav-1 (siCav-1). Antibody against β-Actin was used as loading control.

As mentioned, Cav-1 seemed to be an important regulator of autophagy, and autophagy has an intriguing role in the onset of senescence. Therefore, we decided to explore the relationship between autophagy induction and SIPS induced by Cav-1 downregulation. For this reason, early passage WJ-MSCs were treated either with siRNA against Cav-1 or with siRNA against Cav-1 along with siRNA against the autophagy key molecule, Atg5. Figure 8A,B shows the characteristic downregulation of the levels of Atg5 when cells were treated with the specific siRNA against it. Importantly, as shown in Figure 8C,D there was a striking decrease in the amount of SA-β-gal positive cells when both proteins (Cav-1 and Atg5) were downregulated in early passage cells as compared with the amount of senescent cells in only siRNA against Cav-1 or Atg5 treated cells.

In order to further explore on this connection with autophagy, we analyzed basic molecules involved in autophagy (namely ULK1, Atg13, PI3K-CIII, Beclin-1, Atg5, p62, LC1-I/II) (Figure 8E). The analysis of the protein levels of these molecules in control siRNA treated cells and in siRNA against Cav-1 treated cells revealed that the downregulation of Cav-1 resulted in the induction of the levels of ULK-1, Atg13, PIK3-CIII, Beclin-1 and Atg5, indicating an induction in the autophagic pathway. Moreover, the decrease in p62, combined with the increase in the levels of LC1-II, was indicative of active autophagic degradation [45]. Taken together, the above observations demonstrate the necessity of autophagy induction for SIPS to occur due to Cav-1 downregulation.

## 4. Discussion

Our group recently reported that the inability of OA chondrocytes to repair oxidative stress induced DSB [46] is linked to the failure of Cav-1 spatiotemporal regulation [28]. Specifically, it was shown that Cav-1 is overexpressed in OA chondrocytes as compared to control cells, and that is localized not only at its proper localization sites (membrane/cytoplasm), as in control cells, but also in the nucleus. This resulted in the failure of OA cells to properly upregulate and translocate Cav-1 to the nucleus under stress, as observed in cells from healthy individuals, which could account for their impaired DNA damage response. This was further supported by the fact that, as we showed in the same study, pre-treatment with filipin, a chemical inhibitor that impedes caveolin-1 translocation, results in the inability of cells to repair OS-induced DNA damage [28]. We speculated that this inability of OA cells to properly regulate Cav-1 was due to their senescent status, as demonstrated by us and others [28,44,47,48,49,50]. In order to further explore this assumption, we decided to attempt to clarify the role of Cav-1 in WJ-MSCs as they reach senescence. Therefore, in the current report, we demonstrated that Cav-1 is upregulated, phosphorylated, and translocated to the nucleus in early passage WJ-MSCs when DNA damage is induced from exogenous oxidative insult, before returning to basal/nonphosphorylated levels and exporting the nucleus upon recovery. But, as cells reach senescence or when SIPS is induced, this regulation is lost, as levels of Cav-1/phosphorylated Cav-1 are already increased, and the protein is also localized in the nucleus, even in the absence of any kind of treatment/stress. We speculate that Cav-1 upregulation/translocation to the nucleus in older cells represents the Cav-1 response to DNA damage accumulating due the high levels of OS that cells acquire as they reach senescence, as also demonstrated here. As a result, upon exogenous stress, there was no Cav-1-associated response (de novo upregulation/phosphorylation followed by translocation to the nucleus), which is consistent with the inability of older cells to restore DNA damage. In accordance with this, the downregulation of Cav-1 by specific siRNA treatment in young cells resulted in the persistence of OS-induced DSB that could result in the onset of senescence. An even higher percentages of SA-β-gal, positive cells were present when Cav-1 downregulation was combined with induction of SIPS, as compared with the percentage of senescent cells after SIPS induction alone. Taken together, the above results confirm our previous suggestion regarding Cav-1 involvement in DNA damage response (DDR) that could lead to senescence, in order to prevent the hazardous consequences of genomic instability, either because the damage is extensive or because Cav-1 is absent/unable to perform its homeostatic function in DDR. Furthermore, we report here that the onset of senescence in cells where Cav-1 is downregulated is mediated by autophagy induction, since downregulation of the autophagy key molecule Atg5 simultaneously with Cav-1 downregulation inhibited SIPS. Based on all the previous observations, we propose a unifying scheme that overcomes the apparent discrepancy of published articles where both upregulation [51,52,53,54] and downregulation [31,32,33,34] of Cav-1 have been found to induce senescence.

It has been previously reported in numerous articles (including those reviewed here [19]) that Cav-1 upregulation in various types of cells entering replicative senescence [51,52,53,54], as well as in tissues from aged animals [55,56,57], provides proof of its prosenescent role. Our previous results on OA chondrocytes demonstrating the upregulation of the expression of Cav-1, together with similar observations here in senescent WJ-MSCs, strengthen the validity of the proposed prosenescent function of Cav-1. Furthermore, data on the role of Cav-1 in stress-induced premature senescence (SIPS) have been accumulating over the last 20 years. Volonte and colleagues were the first group to demonstrate that subcytotoxic levels of H_2_O_2_ upregulated Cav-1 expression and induced premature senescence in fibroblasts [58]. As mentioned, we have previously shown that this upregulation occurs in chondrocytes [28] and also in early passage WJ-MSCs under similar stress conditions, as reported here, while also providing evidence linking this upregulation with Cav-1 phosphorylation and subsequent translocation to the nucleus. We also provide data linking both post-translational modification and nuclear translocation to oxidative stress-induced DNA damage response. The latter was proposed recently by Hossain et al. [59]; specifically, the authors showed that the translocation of the membrane-bound tyrosine kinase receptor TIE2 into the nucleus and its involvement in the nonhomologous end-joining repair pathway depend upon Cav-1 phosphorylation and subsequent TIE2/Cav-1 complexes nuclear trafficking.

Importantly, it has been reported that the downregulation of Cav-1 in senescent human diploid fibroblasts results in morphological changes to a nonsenescent-like shape [47], further supporting the notion that an increase in Cav-1 expression levels is necessary for the establishment of senescence. However, the latter observation does not provide any possible mechanistic insight into how silencing of Cav-1 can overcome the telomere shortening occurring during replicative senescence. Furthermore, even though enough evidence has been provided regarding the reversal of the senescence-associated morphological characteristics in cells in which Cav-1 has been silenced, evidence is lacking that links Cav-1 silencing to reversal to a younger functional status. Additionally, the lack of a clear mechanistic scheme regarding the relationship between Cav-1 upregulation and senescence, there are also reports that contradict the above observations, i.e., several articles have shown that downregulation of Cav-1 can also induce senescence via mitochondrial dysfunction [31,32,33,34]. Furthermore, a recent study showed that the downregulation of Cav-1 promotes cellular senescence through the induction and formation of the primary cilium following proteasomal-dependent degradation of aurora kinase A [32]. Based on the latter, Volonte and Galbiati [19] provided an explanation for these obvious contradictions, stating the following: overexpression of Cav-1 contributes to the establishment of replicative senescence and SIPS following exposure to stress while Cav-1 deficiency induces SIPS by promoting primary cilium formation and mitochondrial dysfunction.

Our results add a parameter that provides novel insights to the regulatory scheme described above: we demonstrated that Cav-1 dysregulation/deficiency induces senescence, possibly via cell failure to properly respond to oxidative DNA damage occurring spontaneously. We base this assumption on (a) our previous reported results, showing that by impeding Cav-1 translocation to the nucleus, DSB could not be resolved [28], (b) the fact that late-passage cells already have Cav-1 elevated/phosphorylated/translocated to the nucleus, due to a considerably elevated oxidative status, that could result in elevated OS-induced DNA lesions in older cells, and (c) the downregulation of Cav-1 results in the persistence of OS-induced DSB. In accordance with this, as shown in previous studies with Cav-1^null^ mice, downregulation of Cav-1 could also be responsible for OS induction in the cells, as redox homeostasis is altered, possibly reflecting the role of Cav-1 in mitochondrial function [60,61,62]. Interestingly, these studies not only demonstrated that Cav-1 silencing results in a striking increase in the mitochondrial production of oxidant species, but also in a concomitant activation of autophagy [62].

Autophagy is a regulated catabolic pathway that promotes lysosome-mediated degradation of proteins and organelles, activated under starvation conditions or in stressed cells. The stress-induced autophagic response following Cav-1 knockdown observed here could either reflect the need of cells to generate essential intracellular molecules or to discard OS-induced damaged macromolecules, both of which are essential for maintaining cell homeostasis. A number of recent studies have demonstrated that Cav-1 plays an essential role in regulating autophagy [63,64,65,66]. Furthermore, it was shown that the phosphorylation of Cav-1 is essential for its interaction with Beclin-1 and the activation of autophagy in response to exogenous oxidative stress [67]. Here, in agreement with these results, we showed that Cav-1 downregulation resulted in an induction of autophagy, as indicated by the upregulation in the protein levels of basic autophagy molecular mediators in cells treated with siRNA against Cav-1. The latter could imply the involvement of the autophagic response in SIPS induction after Cav-1 downregulation. This was actually evidenced, when downregulation of Cav-1 was combined with downregulation of the levels of Atg5, and SIPS was inhibited. It should be mentioned that the literature regarding the relationship between autophagy and senescence is still inconclusive, with some reports suggesting that autophagy suppresses cellular senescence by removing damaged macromolecules or organelles, and others indicating that autophagy promotes cellular senescence by facilitating the synthesis of senescence-associated secretory phenotype molecules [68,69]. A recent study provided a possible explanation for this apparent discrepancy: the basic autophagic response has a well-established antisenescence role by eliminating damaged macromolecules/organelles that could induce the senescent phenotype, while selective autophagy of multiple regulatory components that are involved in several senescence-related processes explains its prosenescent function [70]. Our data here add novel elements to the further elucidation of this proposal.

Taken together, our results provide useful knowledge regarding the pleiotropic function of Cav-1 in regulating cellular senescence. We have demonstrated that under either replicative senescence or SIPS, Cav-1 is upregulated in order to respond to OS-induced DNA damage. Additionally, evidence is provided that suggests that when Cav-1 is downregulated, SIPS is induced, possibly due to mitochondrial dysfunction that leads to OS, and subsequently, to oxidative DNA damage. We also showed that the induction of SIPS in the absence of Cav-1 seems to be mediated by autophagy stimulation, which adds insights to the so far poorly understood prosenescent function of autophagy.

Finally, we believe that the current work provides further understanding regarding the senescence of MSCs, which could contribute to the development of strategies that will overcome the senescence-associated impairment of their stemness potential and, therefore, prove significant for regenerative medicine applications.

## Data Availability

Not applicable.
